# What should inpatient psychological therapies be for? Qualitative views of service users on outcomes

**DOI:** 10.1111/hex.13889

**Published:** 2023-10-12

**Authors:** Ceri Morgan, Lucy Clarkson, Rebecca Hiscocks, India Hopkins, Katherine Berry, Natasha Tyler, Lisa Wood, Pamela Jacobsen

**Affiliations:** ^1^ Department of Psychology University of Bath Bath UK; ^2^ Division of Psychology and Mental Health University of Manchester Manchester UK; ^3^ Department of Research and Innovation Greater Manchester Mental Health NHS Foundation Trust Manchester UK; ^4^ Rawnsley Building Manchester Royal Infirmary Manchester UK; ^5^ NIHR School for Primary Care Research University of Manchester Manchester UK; ^6^ Division of Psychiatry University College London London UK; ^7^ Research and Development Department North East London NHS Foundation Trust, Goodmayes Hospital Ilford UK

**Keywords:** acute, inpatient, mental health, outcomes, patient‐reported outcomes, psychological therapies, service users, views

## Abstract

**Background:**

There is limited research on what, when and how outcomes should be measured in psychological therapy trials in acute mental health inpatient wards.

**Objectives:**

This study aimed to consider what outcomes service users think are important to measure.

**Methods:**

This qualitative study explored the views of 14 participants, who had an inpatient admission within the last year, on outcomes of psychological therapies using semistructured interviews. Data were analysed using thematic analysis from a critical realist perspective with both inductive and deductive coding.

**Results:**

The 126 outcomes that were important to participants were mapped onto an established taxonomy of outcomes across different health areas and the socioecological framework to consider the wider context and help summarise the outcomes. Most of the outcomes were mapped to the intrapersonal and interpersonal level. In addition to the outcome mapping, three themes were constructed from the qualitative data: (1) I am not a problem I am a person, (2) Feeling cared for and loved, (3) What does getting better look like.

**Conclusions:**

Our results highlight the need for patient‐reported outcomes which are cocreated with service users, disseminating research and training on preventing dehumanising experiences, enhancing psychological safety and therapeutic relationships and improving access to psychological therapy.

**Patient or Public Contribution:**

The wider People with Personal Experience Involvement Committee at the University of Bath were consulted which included a focus group during the early planning stages. We also collaborated with a person with personal experience, at every stage of the research. This included developing our research question and aims, protocol, participant documents (e.g., information and debrief forms), advertisement and recruitment strategy, interview topic guide, the codes, the final themes and quotes and reviewing the manuscript. People with lived experience of being admitted to an acute mental health inpatient ward participated in our study.

## 1 INTRODUCTION

There is an urgent need to improve the evidence base for psychological therapies delivered in acute mental health inpatient settings, as currently, access is low and varied across settings.^1^, ^2^, ^3^ The National Health Service (NHS) Long Term Plan^4^ outlined that improving access to psychological therapies and crisis care needs to be prioritised. Developing the evidence base for inpatient therapies is an important part of improving access, however, there is a lack of consensus about how to assess treatment effectiveness and what the treatment target should be. Symptom reduction is the primary outcome measure typically reported in studies of psychological therapies delivered in acute mental health inpatient settings, but there is ongoing debate about whether therapies should focus more on supporting people to recover and stay well after discharge.^5^ For example, Wood et al.^5^ proposed that outcomes regarding reducing risk and readmission and increasing safety, which are generally the aims of an acute crisis admission, need exploration and Paterson et al.^6^ highlighted that patient‐centred outcomes such as quality of life and recovery might be important. Additionally, symptom‐focused outcomes without consideration of broader recovery goals or quality of life do not align with the Recovery Model^7^, ^8^ which is often endorsed by adult mental health services. This model proposes that services should support people to regain control and lead a meaningful life and argues against primarily focusing on symptom reduction or management.

The view of service users in determining what outcomes to measure is important but has been neglected so far. People experiencing severe mental health difficulties who are admitted to acute inpatient settings often come from marginalised groups^9^, ^10^ and a large proportion (44%) of adult acute admissions involve detentions under the Mental Health Act.^11^ Also, service users often experience moderate to severe levels of emotional distress and mild cognitive impairment.^12^ This might lead to knowledge produced by service users being questioned due to a credibility deficit and epistemic injustice (where someone is unfairly assumed to be an unreliable knower or is unable to add to, and therefore access, concepts that make sense of their experience within mainstream society)^13^, ^14^ can transpire. Also, ideological power, which operates when people's thoughts, beliefs and feelings are manipulated, ignored or disbelieved, and alternative interpretations are offered or imposed,^15^ can occur.^13^, ^14^ This results in service users' voices not being acknowledged, listened to, or consulted and enhances the possibility of retraumatisation.

Involving a range of stakeholders in research is important as there is a disparity between the views of service users, carers and families and clinicians and researchers.^16^, ^17^ Consequently, without consulting service users, outcomes used in research might not be what service users would consider the most important or relevant.^16^, ^18^ For example, a questionnaire study found that clinicians focused on measuring risk and symptoms while service users were more concerned about involvement and communication.^17^ Disparity can also exist within stakeholder groups. Recent systematic reviews and meta‐analyses have shown that researchers used a variety of outcome measures that measured different symptoms across studies evaluating interventions for people in acute mental health inpatient settings.^5^, ^6^, ^19^, ^20^ Similar to other inpatient mental health core outcome set development studies,^16^ there is considerable heterogeneity in the outcome measures used across studies evaluating inpatient mental health interventions; which makes meaningful comparison of intervention effectiveness difficult. This highlights the need to consider the views of a range of stakeholders and use appropriate methodology to reach consensus.

Within a healthcare context, qualitative research is often conducted to help understand people's experiences and perspectives, and many such studies have been conducted within an inpatient setting to understand service user experiences. For example, qualitative research has been conducted to explore service user experiences of receiving inpatient care.^21^, ^22^, ^23^ Another qualitative study, which explored outcomes for interventions to improve discharge from mental health inpatient services, collated the views of stakeholders via a systematic review and qualitative survey.^16^ However, there is currently no qualitative research exploring what outcomes service users think are important to measure in psychological therapies delivered in acute inpatient settings. Service users' views have historically been ignored; therefore, it is crucial to involve people with personal experience (PPE) in mental health research to stop perpetuating injustice in which marginalised groups are unable to contribute equally to a shared understanding of their experiences.^24^

This paper aims to identify and define what service user perspectives are on outcomes that should be measured in psychological inpatient services. Therefore, the research question is: What do service users think are important outcomes of psychological therapies delivered in acute inpatient settings?

## 2 MATERIALS AND METHODS

### 2.1 Design and ethical approval

The design of the study was qualitative, collecting data via semistructured interviews. This report has been written following the Consolidated Criteria for Reporting Qualitative Research.^25^ The study was conducted as part of a larger project to develop a core outcome set for psychological therapy trials on acute inpatient wards (preregistered protocol on the Open Science Framework available here: https://osf.io/eny8g/). Ethical approval for the qualitative study was included in the wider project approval granted by the Psychology Research Ethics Committee at the University of Bath (Reference number: 22‐017 date of approval: 10 March 2022). All participants gave written consent.

### 2.2 Participants and recruitment

Participants were eligible for inclusion in the study if they were (i) adults aged 18 years old and above, (ii) self‐identified as having been admitted to an inpatient mental health ward (NHS and/or private) in the United Kingdom within the last year (any length of stay), and (iii) were not a current inpatient. Additionally, participants had to be able to read and speak English well enough to understand the study materials and to take part in the interview and were available to be interviewed either over video call or telephone.

Purposive and snowball sampling was used to recruit participants from nonhealth service settings such as self‐help groups and third‐sector organisations, including via social media and charity research recruitment platforms. Third‐sector groups were contacted to support the recruitment of ethnic groups who are over‐represented in acute mental health inpatient settings.^9^ Participants were offered a 15‐pound high street voucher for participating in the interview.

### 2.3 Procedure

The interview schedule was designed and piloted with a person with personal experience researcher (L. C.) and included questions adapted from a previous core outcome set study^16^ (see supplementary materials for interview schedule). The interview began with a brief standardised explanation of what was meant by psychological therapy, and by the term ‘outcome’, and then checking if this made sense to the participant. Interview questions explored the overall subject of: if a psychological therapy was effective, how would we be able to tell, and what sort of things should change in a positive way if psychological treatment ‘worked’.

Interviews were conducted remotely by either video call or telephone and audio‐recorded for transcription. The interviews were conducted by either a trainee clinical psychologist (C. M.) or master's student (R. H.) who had received training in qualitative interviewing and were supervised by the senior author (P. J.). Participants were given the chance to take a break or stop at any time in the interview. We emphasised that the focus was on people's views on outcomes in general, rather than asking people for specific details about their own mental health difficulties or treatment. Participants were therefore free to relate their answers to their own personal experiences but could also choose to talk about things in more general terms if they preferred. Interviewers checked in with participants at the end of the interview to see if they had any concerns or worries, and to check their emotional state and wellbeing before ending the interview. All participants received written debrief forms with signposting information for additional sources of support if needed.

### 2.4 Theoretical framework and analysis

Data were analysed from a critical realist perspective therefore analysis assumed that the participant's lived experience was real while also acknowledged the inability of researchers to entirely access that reality. We used two main approaches to analysing the data. First, we wanted to capture the range and types of outcomes service users thought were important to measure in psychological therapies delivered in acute inpatient settings. To do this, we used a primarily deductive approach, using a coding frame based on an established taxonomy of outcomes across different health areas, including mental health.^26^ The taxonomy has five core areas (death, physiological/clinical, life impact, resource use and adverse events) further divided into 38 more detailed outcome domains such as physical functioning, need for further intervention and psychiatric outcomes. This taxonomy was chosen because it includes a range of domains to help categorise, summarise and reflect service user led outcomes. We agreed that if outcomes did not fit this framework a new domain would be clearly reported. We then considered how these different types of outcomes (e.g., *psychiatric symptoms, functioning, resource use*) were positioned within a wider socioecological framework^27^ and we mapped the outcomes and taxonomy domains to this framework. We recognised that there would be interactions between and within the different levels (e.g*., intrapersonal, interpersonal, community*) and that the mapping could be malleable.

Second, we wanted to capture the broader context of the meanings and importance of these outcomes from a lived experience perspective. For this we used a primarily inductive approach, using reflexive thematic analysis.^28^

### 2.5 Coding process and how themes were identified

Interviews were transcribed verbatim and checked by both the interviewers (C. M. and R. H.). C. M. led the data analysis by developing the initial coding frameworks after a period of deep immersion in the data (e.g., many hours conducting the interviews, listening to recordings, checking, reading and rereading of transcripts). Data analysis software NVivo (March 2020 release) was used to help the coding process. Following coding, a thematic map was created and then shared and further refined in an iterative process with the wider research team. The final thematic map for both the inductive and deductive analysis were checked and agreed by the research team.

### 2.6 Research team and reflexivity

The core analysis team consisted of C. M. (trainee clinical psychologist), R. H. (master's student), P. J. (clinical psychologist) and L. C. (person with personal experience researcher). The analysis team all identified as female, White British, and were in the 20s–40s age range. C. M. and P. J. had previous experience of working on an acute mental health ward. L. C. had lived experience of receiving care on an inpatient ward. None of the participants were previously known to the interviewers or any members of the wider research team.

To remain aware of their own position and enable bracketing, which is the method of putting aside one's own knowledge and beliefs about the research,^29^, ^30^ C. M. and R. H. kept notes in a reflective diary during and after the interviews and used regular research supervision with the senior author P. J. to reflect on the codes and themes being identified and their own lens. The first author (C. M.) attended antiracism and whiteness training facilitated by the University of Bath to increase their awareness of how the research team's demographics, identity and positionality might affect the interpretation of the data. This was regularly discussed in research supervision and with colleagues from minority backgrounds.

### 2.7 PPE involvement

We collaborated with L. C. at every stage of the research, including developing the research question and aims, protocol, participant documents (e.g., information and debrief forms), advertisement and recruitment strategy, interview topic guide, codes and the final themes and quotes. L. C. led consultation with the wider PPE Involvement Committee which included a focus group during the early planning stages. All PPE members were appropriately reimbursed in line with guidelines at the University of Bath.

## 3 RESULTS

### 3.1 Participants

Fourteen participants took part in the study between May and July 2022. Five participants were aged 18–29, seven were aged 30–39 and two were aged 40–49. Eight participants identified as female, four as male and two did not specify their gender. Three participants additionally shared that they were transgender. Four participants identified as Black, Black British, Caribbean, or African; four as White British; three as mixed or multiple ethnic groups; one as other ethnic groups (Chinese), one person did not disclose their ethnicity, and one response was missing due to it being inaudible on the recording. The interview durations ranged from 16 to 39 min, and all were conducted via video call.

### 3.2 Deductive coding of outcomes

**Figure 1:**
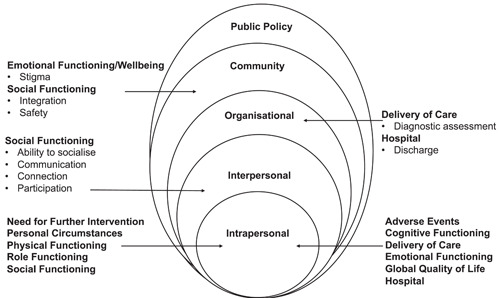
Diagram of the reported outcomes mapped onto the taxonomy outcome domain^26^ and socioecological framework.^27^

Participants referred to a total of 126 different outcomes that were important to them during the interviews. Table 1 outlines the outcomes mapped to the taxonomy domain.^26^ The emotional functioning and psychiatric outcomes domains had the greatest number of outcomes mapped to them. Figure 1 shows a diagram of the outcomes reported by participants which were mapped onto the taxonomy domain^26^ and each level of the socioecological framework.^27^ The taxonomy domains relate to the following core areas: physiological/clinical, life impact, resource use and adverse events.^26^ No outcomes were mapped to the outcome domain of mortality/survival and core area of death.

**Table 1:** Summary of outcomes important to participants mapped to the taxonomy domains^26^ and intrapersonal level of the socioecological framework.^27^

Adverse events (7)	Cognitive functioning (6)	Delivery of care (13)	Emotional functioning (25)	Global quality of life (5)	Hospital (3)	Need for further intervention (3)	Personal circumstances (3)	Physical functioning (13)	Psychiatric outcomes (23)		Role functioning (2)	Social functioning (1)
Incidents (4)	Attitudes and beliefs (4)	Engagement (5)	Coping techniques (8)	Quality of life (5)	Hospital use (3)	Relapse (3)	Living situation (3)	Physical health (7)	Mental health (6)		Attendance (1)	Feeling supported (1)
Self‐harm (3)	Planning skills (2)	Treatment satisfaction (4)	Hope (5)					Activity (3)	Anxiety (5)		Work status (1)	
		Medication adherence (2)	Perceived control over condition (2)					Self‐care (2)	Mood (5)			
		Medication satisfaction (1)	Stress (4)					Sleep (1)	Depression (4)			
		Assessment (1)	Empowerment (1)						Symptom reduction (2)			
			Goals (1)						Distress (1)			
			Identity (1)									
			Insight (1)									
			Self‐efficacy (1)									
			Self‐esteem (1)									

#### 3.2.1 Intrapersonal characteristics

The participants identified 104 outcomes which related to intrapersonal characteristics. These outcomes were mapped to the following taxonomy domains^26^: adverse events, cognitive functioning, delivery of care, emotional functioning, global quality of life, hospital, need for further intervention, personal circumstances, physical functioning, role functioning and social functioning.

#### 3.2.2 Interpersonal processes

Participants identified 14 outcomes related to interpersonal processes. These outcomes were mapped to the social functioning taxonomy domain. The outcome descriptions included the ability to socialise (2), communication (7), connection (4) and participation (1).

#### 3.2.3 Organisational factors

Four outcomes connected to organisational processes were mapped to delivery of care (diagnostic assessment, *n* = 1, specific scale for condition, *n* = 1) and hospital (discharge *n* = 2) taxonomy domains.

#### 3.2.4 Community factors

There were four outcomes reported by participants which related to community factors. These outcomes which were mapped to the emotional functioning or wellbeing (stigma, n = 1) and social functioning (integration, *n* = 2 and safety, *n* = 1) taxonomy domains.

#### 3.2.5 Public policy

Participants did not identify any outcomes which related to public policy.

### 3.3 Inductive coding of outcomes

**Figure 2:**
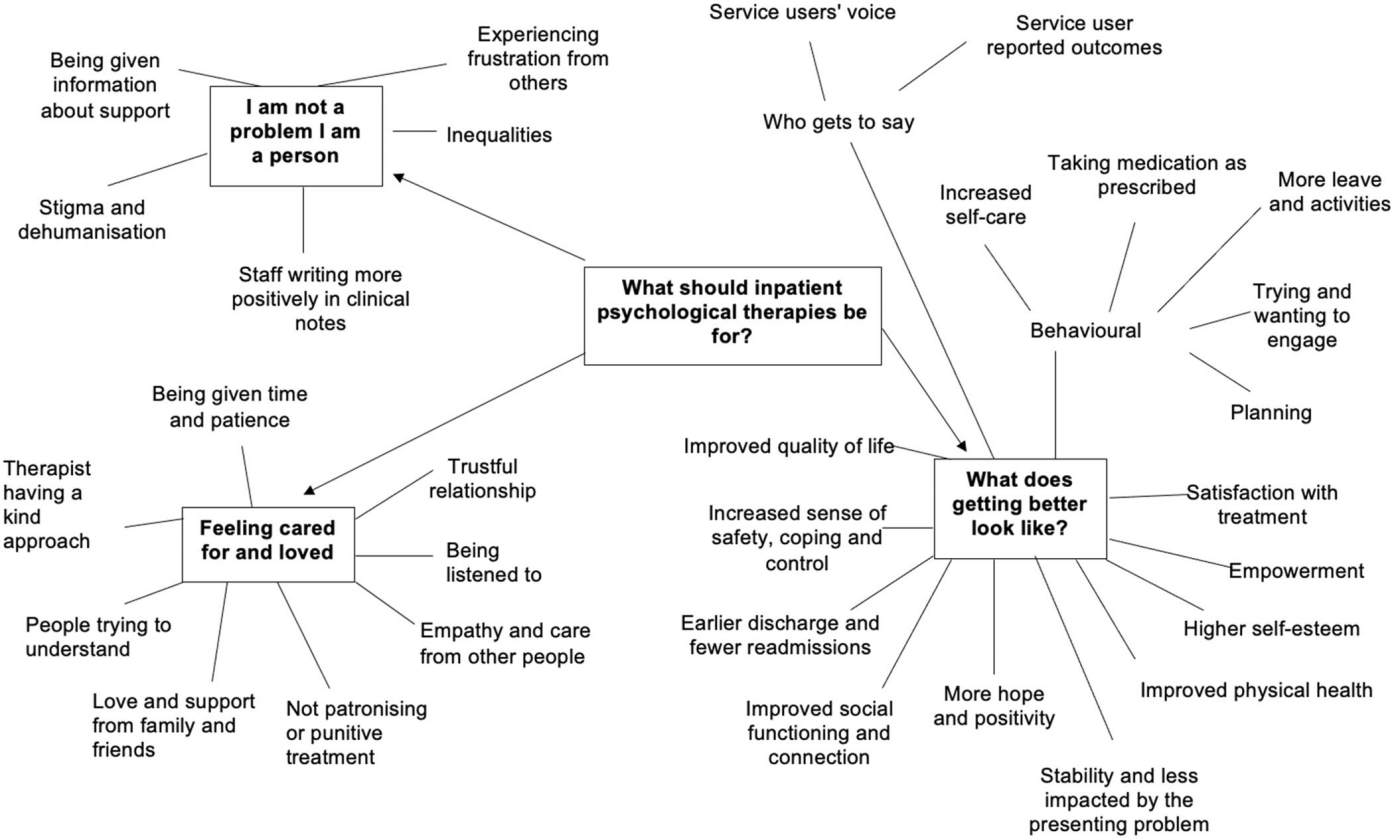
Thematic map.

From our inductive analysis of the wider context of why these outcomes were important to service users, we identified three themes. Figure 2 shows the thematic map which outlines the themes and codes.

#### 3.3.1 Theme 1: I am not a problem I am a person

As participants outlined outcomes which were important to them to measure in psychological therapies delivered in acute inpatient settings, they shared experiences of staff becoming frustrated with them and reflected on experiences of stigma, metadehumanisation and inequalities such as unequal and inconsistent provision of psychological therapy while staying on the acute mental health ward. Metadehumanisation is the perception that oneself is viewed as less than human by other people.^31^ These experiences influenced self‐image, self‐worth and ultimately developing an internalised sense of being a problem rather than a person. Experience of frustration from healthcare staff could reinforce self‐critical thoughts and have a negative impact on mental health.

Most people make it very clear that they're [obscenity] off with you and you're frustrating them and they can't be bothered to deal with you. (Participant C)

Participants recalled that the way they were spoken to was important and influenced whether they thought other people saw them as a person.

Talking to me like (.) you know I'm not a [obscenity] ((laughs)) like just as if like I'm a normal person. (Participant C)

Participants shared that certain diagnoses such as emotionally unstable personality disorder were stigmatised and penalised which was depersonalising as people were being seen as a diagnosis rather than an individual. Furthermore, accounts of being misgendered and noticing age and racial inequalities suggested the lack of person‐centred care and universal human rights being respected. Experiences of always being watched and staff writing negative clinical notes left participants wanting to be treated as a person and with positivity. Participants referred to power imbalances, and it was apparent that participants experienced epistemic injustice as their views were not seen as credible, and they were dismissed because of their mental health difficulties. Service users deserve to be treated humanely and be offered the opportunity to report their own outcomes, but the above experiences might be a barrier to this.

Being dismissed as a psychotic. (Participant A)

#### 3.3.2 Theme 2: Feeling Cared for and Loved

Participants reported that they valued staff being kind, patient, empathic and caring. Many shared that being listened to and being given time was fundamental to enabling them to feel cared for and better about themselves and improve their wellbeing.

The only thing really that that's helped me is is those like people just being nice like by just like empathising and just like listening to me. (Participant C)

Wanna build that like trustful relationship with the psychologist that's involved. (Participant C)

I felt like they really did care it was like the first time (.) like anyone actually cared ((laughs)) umm ((pause)) and I guess it just made me feel (.) better about myself. (Participant D)

Participants accounts suggested that therapeutic relationships and the concept of psychological safety should be both a precondition and an outcome of psychological therapy. Staff trying to understand assisted psychological safety to engage in therapeutic work while building psychological safety is an outcome of therapy too.

Participants spoke about the significance of the love and support they received from friends and family and how this motivated them. Although when this is not possible, participants suggested that feeling supported and cared for could be provided by anyone.

They were always there to listen to me, they made me feel loved. (Participant J)

I feel that every patient (.) does is admitted or (start of condition) needs love wants people to give him reasons for recovering quickly. (Subject N)

#### 3.3.3 Theme 3: What does getting better look like?

Our third theme was also generated as an outcome of the data and research aims. Participants shared that improved physical health, a sense of safety, coping and control were important indicators of recovery. Their narratives highlighted that when someone is getting better you can see various positive behavioural changes such as increased self‐care, engagement in activities and progression of leave. Participants spoke about reduced use of inpatient services, empowerment and more stability. They described a sense of relief, social connection and treatment satisfaction associated with getting better.

I would feel relieved within me. I would I would feel more relaxed. (Subject F)

You can see some smiles on her face and then she just acts happy, she responds to things around her, things that could make her smile and you see her smile and things as things to make her happy. She will have that reactions in their face. (Participant H)

Many participants recalled a shift in perspective, feeling more hopeful and looking more towards the future.

Visualising you're recovering and what you could do. (Subject L)

Participants described how their views on their own recovery and readiness for therapy were often dismissed and overlooked. They emphasised that staff do not know everything. They suggested that it might look like someone is getting better if they are engaging in psychological therapy when they might feel pressured into it to get discharged sooner. This highlights the complexity in outcomes and who is reporting them.

It would just be interesting how willing patients were to do the therapy because I think sometimes we are kind of bullied into it … do they feel pressured to take part in it, right? Is it something that they actually (.) especially if you're (in)voluntary and part of the thing is like do what they tell you and you can get out and then you're doing these sessions and um you really don't want to. (Participant A)

Participants proposed that service users are in the best position to provide true insight into the reality of their experiences and recovery. The narratives indicated that service users should be enabled to provide their view on outcomes and the helpfulness of therapy.

The person is actually in charge of you know giving the (response) if anything has changed or not. (Subject H)

Patient observation of symptoms because obviously even though staff are watching you staff don't see everything like a lot of staff are oblivious to lots of things. (Participant E)

## 4 DISCUSSION

This paper describes a qualitative study exploring the views of 14 service users on the outcomes of inpatient psychological therapies. The 126 outcomes that were important to participants were mapped onto a taxonomy of different outcome domains^26^ and different levels of a socioecological framework.^27^ Most of the outcomes (104 outcomes) were mapped to the intrapersonal level, with 14 outcomes to the interpersonal level and four outcomes to the community and organisation level. No outcomes reported mapped to the public policy level. Three themes were created from the semistructured interviews: (1) I am not a problem I am a person, (2) Feeling cared for and loved, (3) What does getting better look like.

### 4.1 Strengths and limitations

An important strength in this study was the involvement of a person with personal experience as part of the research team which enhanced the study design and recruitment, ecological validity and impact and enabled the findings to be more meaningful and relevant to service users, healthcare services and the community.^24^, ^32^, ^33^, ^34^ These benefits support the proposition that services and research should be codesigned with service users.^35^

Participants were all people who had experienced an inpatient admission within the last year, so they were able to share recent experiences which relate to current clinical practice in the United Kingdom. Our inclusion/exclusion criteria did not specify that participants needed to have engaged in any psychological therapy while staying on the acute mental health ward. It may have been difficult to recruit this sample due to the limited amount of psychological therapy currently being delivered in acute inpatient mental health settings. Our sample incorporated diverse ethnicities, ages and gender. This is important as a wide range of different people receive care in inpatient settings, with some groups of people being over‐represented in inpatient settings compared to the general population. For example, there is an over representation of people from ethnic minorities in inpatient care.^9^, ^10^ It is important therefore that the sample of participants reflects the population to which the results would be applied that is, people receiving inpatient care. We acknowledge that our sample is not completely representative as for example South Asians are not represented. Generalisability is not the aim of qualitative research, but we recognise how the characteristics of our participants such as age, gender and ethnicity and the context of our research being conducted digitally may have shaped or modified our findings. Additionally, reflections on our sample size are best informed by ‘information power’,^36^ which focuses on key dimensions such as study aim and sample specificity rather than ‘data saturation’, as this is an inconsistently defined term in qualitative research.^37^, ^38^ Our narrow aim and sample consisting of people who had an acute mental health inpatient admission within the last year suggests relatively high information power.^39^

The interviews were relatively short and telephone interviews were offered as an option to try to minimise digital exclusion issues. Coding was completed by the first author alone which may have limited the range of different lenses through which the data were initially viewed, however other members of the research team also contributed to refinement and development of the final thematic maps which increased the overall range of input into the analysis process. The researchers held a critical realist perspective which enabled them to recognise that human practices influence findings and the interpreted realities, and the researchers are a part of the world they aim to understand.

### 4.2 Comparison with existing literature

This study adds to the literature by outlining the outcomes that are important to service users. Most outcomes were mapped to the intrapersonal and interpersonal level of the socioecological framework.^27^ This supports research which suggests that psychological interventions can help people make sense of a crisis and lead to changes at an interpersonal and intrapersonal level.^1^ Our findings indicate that there might be interactions between and within the systems as suggested by previous literature.^40^ For example, outcomes which related to the social functioning taxonomy domain^26^ mapped to the intrapersonal, interpersonal, community levels of the socioecological framework.^27^ Also, outcomes related to the delivery of care taxonomy domain^26^ mapped to the intrapersonal, interpersonal, organisational levels of the socioecological framework.^27^ This suggests that outcomes need to be understood beyond the individual.

We noted that participants did not generate any outcomes which mapped onto the public policy level, which could have arisen from the nature of the questions in the topic guide which did not necessarily prompt people to consider outcomes which mapped onto levels other than the intra‐ and interpersonal (community, organisational and public policy). Commissioning of services and psychological therapy is an example of an outcome which might be mapped onto the public policy level. This study was based on people who had experienced inpatient care in an acute setting in the United Kingdom which has an individualist culture with a preference to focus on the individual.^41^ Perhaps in other cultures outcomes would be distributed differently across the socioecological framework and changes might be less focused on the individual. This also highlights the importance of including a range of stakeholders in generating core outcome sets, as different groups may focus on different levels of outcomes (e.g., commissioners may be more focused on policy‐level outcomes compared to service users and clinicians).

In line with previous research, our study highlights that service users experienced metadehumanisation and epistemic injustice while staying on acute mental health inpatient wards and their view was deemed as not credible.^14^, ^42^ Participants shared that their views were not listened to. This is in line with other research where service users felt their needs were dismissed and they were not being ‘heard’.^13^ These experiences perpetuate and add to power imbalances and the powerlessness often experienced by service users. Participants desiring to be treated as a person replicates previous qualitative studies in acute mental health inpatient settings which found that a ‘human’ relationship was important.^43^

Participants shared that getting better involves developing a sense of safety and feeling loved and cared for which is understandable when the experience of being an inpatient on a mental health ward can feel unsafe and distressing.^21^, ^22^ Phillips et al.^44^ described how service users experienced a lack of prioritisation of the therapeutic relationship despite this being fundamental to them. Maslow's Hierarchy of Needs outlined that safety needs to be sought before attempting to meet any higher‐level needs.^45^ Participants communicated that psychological safety was an important outcome and precondition for therapy. McAndrew et al.^46^ explored the importance of therapeutic relationship in acute inpatient mental health settings. Participants' views aligned with previous literature which suggested that service users value: being listened to, a trusting relationship with staff and positive attitudes.^46^

Participants spontaneously sharing that they experienced unequal and inconsistent provision of psychological therapy while staying on the acute mental health ward is concerning and confirms that barriers exist,^1^, ^47^, ^48^ despite national guidelines^49^ and the 5‐year forward view for mental health^35^ endorsing that evidence‐based psychological interventions can be started in acute inpatient settings and recognition of the value.^50^, ^51^ Our findings support studies which emphasised that service users have said they need improved access to psychological therapies^23^, ^52^ and service users value the provision.^53^

Participants views about outcomes included improved physical health. This corresponds with literature which highlights the importance of physical health monitoring on mental health wards which has historically been overlooked^54^, ^55^ and addressing large health inequalities for people with severe mental ill health.^56^

### 4.3 Clinical implications and future research

The findings of this study support the need for service user‐reported and service user‐generated outcome measures.^57^, ^58^ Developing a core outcome set that incorporates all stakeholders' views is an important next step in this area of research. It would be interesting to reflect on other stakeholder's views. Additionally, professionals and services need to ensure that service users can report on their own desired outcomes and their voice will be heard.

Our study supports recommendations by Bacha et al.^42^ that mental health services and professionals need to be aware of the relational components of safety, power and identity and how service users can feel dehumanised and disempowered. It is important that research is disseminated, and training is delivered to widen people's awareness and understanding of epistemic injustice, dehumanisation and inequalities in inpatient settings. Additionally, supporting clinicians to develop therapeutic relationships with services users (by being kind, patient, empathic, caring and taking time to listen) to enable service users to feel cared for and safe needs to be prioritised.

As specified in the NHS Long Term Plan,^4^ improving access to psychological therapies and crisis care is needed. The barriers to accessing psychological therapy in acute mental health inpatient settings including feasibility in NHS care pathways, environmental constraints,^1^ brief admissions and high turnover of staff need to be overcome to ensure the provision of therapy is consistent.

## AUTHOR CONTRIBUTIONS

Ceri Morgan led the design of the study in consultation with the rest of the authors. Ceri Morgan and Rebecca Hiscocks conducted the qualitative interviews. Rebecca Hiscocks transcribed the interviews and Ceri Morgan edited them. Ceri Morgan, Rebecca Hiscocks, Lucy Clarkson, India Hopkins and Pamela Jacobsen contributed to data interpretation. Ceri Morgan led the analysis and wrote the initial draft of the manuscript and all authors contributed to editing and finalising the manuscript and read and approved the final version for submission.

## CONFLICT OF INTEREST STATEMENT

The authors declare no conflict of interest.

## Supporting information

Supporting information.

## Data Availability

The data that support the findings of this study are openly available in the University of Bath repository (https://doi.org/10.15125/BATH-01265).
