# Supplementary material for: What should inpatient psychological therapies be for? Qualitative views of service users on outcomes
Source: Health Expect. 2023 Oct 12;27(1):e13889. doi: 10.1111/hex.13889 (PMC10726158; doi:10.1111/hex.13889)
Supplement: Supplementary file 1 — Supporting information. [file HEX-27-e13889-s001.docx]

**Supplementary Materials**

Interview Schedule for Main Research Project

**Development of a Core Outcome Set for psychological therapy trials in acute mental health inpatient services**

Semi-structured Interview Topic Guide – Service Users
(prompts listed below the question)

***Introduction***Thank you for agreeing to talk to me today. I would like to take a moment to introduce myself and to recap what the interview will involve. My name is [insert name of interviewer], and I am conducting this study for my Clinical Psychology Doctorate at the University of Bath. I am going to ask you a few general questions about topics such as what you think might be important to measure in future inpatient research, and how we would be able to tell if an inpatient therapy had worked well. You will not be asked any specific questions about your own personal experiences of inpatient care; we are just interested in your general views and opinions.

It is important to remember there are no right or wrong answers. I am really interested in your experience and your opinions, whatever they may be. We can pause or stop anytime you need to, or if you prefer not to answer a question, please just let me know and we can move onto the next question. You do not have to talk about anything you do not want to. We expect this interview to take around 30 - 60 minutes, depending on how much you would like to say. Does that sound okay? Is there anything you want to ask me about the interview at this point before we start?

If there are any connection issues, I will try to call you again, and if that does not work, I will email you. Is that okay?

After completing the interview, you can choose to be sent a £15 high street voucher such as ‘Love2shop’ to thank you for taking part. This voucher can be sent to you via email or post.

It is difficult to compare results from different projects about which psychological therapies work best because they do not all measure the same outcomes. One solution is to develop a Core Outcome Set which are an agreed set of outcomes which should always be measured and reported as a minimum in all trials (projects) in a particular health area. This project involves developing a Core Outcome Set for trials evaluating psychological therapies on acute mental health inpatient wards. There are various parts to this research and this stage involves completing an interview.

First, we will begin with some questions about yourself. Please only choose one option when answering and if you prefer not to answer a question, we can move onto the next question. Does that sound okay?

**Participant Demographic Details**

1) What is your age?
18-29 □ 30-39 □ 40-49 □ 50-59 □ 60 or older □

2) How would you define your gender?
Man □ Woman □

I identify my gender as: …

3) How would you define your ethnicity?
Asian or Asian British □ Black, Black British, Caribbean or African □ Mixed or multiple ethnic groups □ White □ Other ethnic group □ Prefer not to answer □

Thank you. Now we will talk about what you think might be important to measure in future inpatient research, and how we would be able to tell if an inpatient therapy had worked well. Does that sound okay?

**1) My first question is, if a psychological therapy delivered to someone on an acute mental health ward was effective, how would we be able to tell??**

Define ‘psychological therapy’ and ‘psychiatric/acute mental health ward’

- **Psychological therapy** refers to any psychological therapy with a professional/therapist. Psychological therapies are occasionally referred to as talking therapies. They involve exploring psychological difficulties that might be getting in the way of how we would like to feel. Psychological therapy is a collaborative space to explore difficulties in a safe and confidential setting. Psychological therapies can include, but are not limited to, Cognitive Behavioural Therapy, Mindfulness, Interpersonal Therapy, Psychotherapy, Psychodynamic Therapy, and Systemic/Family Therapy.
- **Psychiatric/acute mental health ward** refers to a ward in a mental health hospital which people are admitted to voluntarily or whilst they are detained under the Mental Health Act.

Prompts/probes:
- Explore any examples

**2) Can you think of any important outcomes to measure in research assessing the effectiveness of psychological therapy for people on acute mental health wards?**

Define ‘outcomes’

**Outcomes:** The measures of a treatment/intervention which reflect any changes or results that occur. For example, in a clinical trial of how well psychological therapy works, ‘outcomes’ might include a quality of life measure or relapse/reoccurrence.

Prompts/probes:

**3) If someone is receiving treatment on an acute mental health ward, what kinds of things should change in a positive way for them if the treatment works?**
Prompts/probes:

*- can you think of any positive changes?*

**4) What do you think is the most important aspect of psychological therapy in an acute mental health ward?**

Prompts/probes:

**5) If you had a psychological therapy during a stay on a mental health ward, what would be the most important things to measure to check whether it had worked or not?**

If not applicable move onto question 6.

**5a)** **Was there any one person/group/intervention which really made a difference for you? If so, what was it? What made it so powerful?**
*Prompts/probes:*

**6) That was all the questions I had for you today. Do you have any other feelings or concerns that have not been addressed in previous questions?**

Thank you very much for taking part in this study. As part of the research, we will put together information from all the different interviews and come up with a summary of themes. Also, just a final reminder that all data from the interview will be stored anonymously, so we will not use your name, and you will not be personally identifiable in any reports or publications from the research.

We can provide you with a copy of the final research report if you are interested in receiving this.
